# Molecular Mechanisms of Memory Consolidation, Reconsolidation, and Persistence

**DOI:** 10.1155/2015/687175

**Published:** 2015-08-26

**Authors:** Emiliano Merlo, Pedro Bekinschtein, Sietse Jonkman, Jorge H. Medina

**Affiliations:** ^1^Behavioural and Clinical Neuroscience Institute and Department of Psychology, University of Cambridge, Cambridge CB23EB, UK; ^2^Instituto de Biología Celular y Neurociencias “Dr. Eduardo De Robertis”, Facultad de Medicina, Universidad de Buenos Aires, CONICET, 1121 Buenos Aires, Argentina; ^3^Department of Pharmacology and Systems Therapeutics, Icahn School of Medicine at Mount Sinai, New York, NY 10029-6574, USA; ^4^Departamento de Fisiología, Facultad de Medicina, Universidad de Buenos Aires, 1121 Buenos Aires, Argentina

In the last decades there have been significant advances in our understanding of the cellular and subcellular mechanisms underlying changes in synaptic connectivity that subserve memory formation. The so called Theory of Synaptic Plasticity and Memory has gathered a wealth of experimental support from different areas of neuroscience to become the main phenomenological description of memory at the behavioural level. This special issue of neural plasticity compiles some of the most recent advances in our understanding of the mechanisms underlying formation and persistence of different types of memories from invertebrates to humans. Contributions from different laboratories around the world pinpoint hot topics in this area of memory research, highlighting growing avenues for future research.

The experience of a salient event can lead to the formation and storage of a long-term memory that can sculpt and alter future behaviour up to a lifetime of an individual. This unique and highly adaptive behavioural capacity relies on specific changes occurring within the brain. Specific signalling pathways and patterns of gene expression are required in neuronal and nonneuronal cells for the stabilization and long-term persistence of synaptic changes that underlie memory. Depending on the retrieval conditions, these fully consolidated memories can undergo reconsolidation or extinction that will maintain or inhibit the expression of the original memory, respectively. These opposing memory processes recruit distinctive subcellular events in order to restabilize the original memory or to form a new inhibitory memory trace. The formation of associative memories and their maintenance are evolutionary conserved phenomena present from the simplest to the most complex animals. The use of a multidisciplinary approach, comprising behavioural, physiological, and molecular analysis, in combination with a variety of wild and laboratory animals, from invertebrates to humans, brings light into the intricate mechanisms of memory. The three research papers and five review articles included here were revised by at least two international experts and their comments helped in making each piece an even more compelling article.

This Issue includes two articles addressing novel mechanisms in memory consolidation. B. Silva et al. used larvae of the fruitfly* Drosophila* to show that muscarinic-type acetylcholine receptors contribute to the generation of olfactory aversive memory. Besides the obvious anatomical differences between vertebrate and invertebrate nervous systems, this article further supports the evolutionary conserved role of key contributors to memory consolidation. T. P. Todd and D. J. Bucci show that retrosplenial cortex (RSC) is specifically involved in forming associations among the neutral stimuli that are present in the environment. Furthermore, they discuss evidence that posits RSC as a site in which multiple cues are linked together in the service of memory formation and persistence after training.

A comprehensive review article by D. Moncada et al. serves both as an introduction and a thorough revision of the existing literature regarding the experimental findings supporting the behavioural tagging process in rodents and humans. This working hypothesis links the concept of synaptic tagging proposed by Morris and Frey in the late 90s with more recent evidence of a significant promoting effect of a novel behavioural experience in the formation of new and independent associative memories. Moreover, M. Tomaiuolo et al. establish novel links between the synaptic tagging hypothesis and memory persistence, showing that a dopamine- and Arc-dependent maintenance tagging process may operate in the hippocampus late after acquisition for the persistence of long-lasting memories. Ending the persistence mechanisms section, J. B. Hales et al. investigated the effect of the zeta-inhibitory peptide (ZIP) in the persistence of recognition memory in rats. This article shows that recent, but not remote, object recognition memories can be disrupted by ZIP infusion into the hippocampus and suggests a dynamic role of hippocampal LTP-dependent mechanisms supporting strong recognition memories shortly after training.

J.-P. Morin et al. discuss at length the role of the protein Arc as one of the main molecular substrates of memory. They go over its characteristics and regulation and the reasons why this molecule could be an essential part of the memory engram. They propose that Arc possesses particular characteristics like its persistent expression after learning its pre- and posttranslational regulation and its interactions with molecules at the synapse that make it an ideal candidate to mediate plasticity in the cells activated by a given learning experience.

Adult neurogenesis in the dentate gyrus of the hippocampus has gained increasing interest as a potential plasticity mechanism for learning and memory at the cell and system level of analysis. The article by S. Yau et al. addresses the role of adult hippocampal neurogenesis in learning and memory focusing on novel findings that indicate a function for this process in two features of memory. One of these features is “pattern separation” which refers to the computational process involved in separating the representations of similar learning experiences. The second is the far less studied process of memory forgetting, which will certainly be one of the new most interesting fields in memory research. The authors incorporate this new information and relate it to treatments such as environmental enrichment and voluntary exercise, which are known to increase neurogenesis.

The issue presents an article dedicated to analyse the implications of memory studies for the development of novel therapeutical tools for the treatment of psychiatric disorders in humans. In particular, C. Köhler et al. propose that the manipulation of the reconsolidation of autobiographical memories might represent a novel therapeutic opportunity for depression treatment. The authors suggest that disruption of memory reconsolidation could serve as a novel approach for the modification of dysfunctional autobiographical memories associated with major depressive disorder.

We are very pleased to introduce this special issue that covers a variety of features of memory at different levels of analysis. The persistent nature of maladaptive memory components is a common characteristic in several psychiatric disorders including posttraumatic stress disorder (PTSD), specific phobias, and drug addiction. We believe that understanding the key molecular mechanisms underlying the formation, persistence maintenance, and forgetting of different forms of memories will prove to be invaluable at both the foundational and translational levels, helping the design and development of new therapeutical approaches.

